# Does Terminology Matter When Measuring Stigmatizing Attitudes About Weight? Validation of a Brief, Modified Attitudes Toward Obese Persons Scale

**DOI:** 10.21203/rs.3.rs-4208912/v2

**Published:** 2024-07-22

**Authors:** Caitlin A Martin-Wagar, Katelyn A Melcher, Sarah E Attaway, Brooke L Bennett, Connor J Thompson, Oscar Kronenberger, Taylor E Penwell

## Abstract

**Objective** Commonly used terms like “obese person” have been identified as stigmatizing by those with lived experience. Thus, this study sought to revise a commonly used measure of weight stigmatizing attitudes, the Attitudes Toward Obese Persons (ATOP) scale. **Methods** The original terminology in the 20-item ATOP (e.g., “obese”) was compared to a modified version using neutral terms (e.g., “higher weight”). Participants ( *N* = 832) were randomized to either receive the original or modified ATOP. **Results** There was a statistically significant difference, with a low effect size ( *d* =-0.26), between the scores of participants who received the original ATOP ( *M* = 69.25) and the modified ATOP ( *M* = 72.85), *t* (414) = -2.27, *p* = .024. Through principal component analysis, the modified ATOP was found to be best used as a brief, 8-item unidimensional measure. In a second sample, confirmatory factor analysis verified the fit of the brief, 8-item factor structure. **Conclusions** Findings suggest a modified, brief version of the ATOP (ATOP-Heigher Weight; ATOP-HW) with neutral language is suitable for assessing negative attitudes about higher-weight people. The ATOP-HW may slightly underestimate weight stigma compared to the original ATOP. Further examination of the terminology used in weight stigma measures is needed to determine how to best assess weight stigma without reinforcing stigmatizing attitudes. The findings of the present study suggest that the use of neutral terms in measures of anti-fat bias is a promising solution that warrants further investigation.

## Introduction

Weight stigma refers to negative attitudes, beliefs, stereotypes, and actions based on a person’s weight, and is pervasive across education, healthcare, media, and the workplace[1,2]. Some weight-normative public health campaigns invoke weight stigma and shame in hopes of motivating people to lose weight[3]. However, rather than promoting health, weight stigma is associated with numerous adverse mental health outcomes, such as disordered eating, depression, anxiety, stress, body dissatisfaction, poor self-esteem, satisfaction with life, and substance use[2,4-10]. Similarly, experienced weight stigma has negative physical health correlates, such as increased cortisol reactivity, avoidance of healthcare, reduced engagement in physical exercise, and arterial pressure[11-15]. Finally, weight stigma is associated with adverse social and academic outcomes, such as rejection by peers and lower classroom engagement[9].

Given the toll of weight stigma, it is important to determine the best ways to measure and address weight stigma. The implications of terminology choice when discussing weight are clear. For people trying to lose weight, the terms “fat,” “obese,” and “extremely obese” used by healthcare providers have been rated as the most undesirable[16]. Further, individuals preferred that providers merely referenced individuals’ weight rather than use terms viewed by individuals with higher weight as stigmatizing[16]. A systematic review of 33 studies elaborates further; researchers found a preference for neutral terminology (e.g., “weight”) over terms like “fat” and “obese” across most of these studies[9]. Children have reported feeling upset that their parents described them as “large,” “fat,” “obese,” and “overweight,”; the participants preferred for their parents to use the terms “healthy weight” or “normal weight”[17]. Despite being consistently described by individuals with lived experience as stigmatizing, most current measures of weight stigma still use the medical language (e.g., “obese” and “overweight”). Most of these measures were developed before the stigma of these terms was well understood, suggesting the language used may be outdated.

Recently, the American Psychological Association recommended using inclusive language when discussing body size and weight[18], which reiterated the points outlined by Meadows & Danielsdottir (2016) that suggest using neutral terms such as “weight” or “higher weight” and suggest psychologists not use person-first language as there is no consensus on whether this is preferred[19-20]. Similarly, the National Institute of Health recommends using “*person with higher weight, higher-weight individual, person with a larger body,* or other similar neutral descriptors, rather than *person with obesity or overweight”*[21]. Clearly, there is a growing movement to modify the terminology used to describe people with higher weight. Perhaps, more terminology changes will support a less stigmatizing environment for those with higher weight.

Besides conveying stigma, the term “obese” lacks a solid definition and meaning. In a position statement from the World Obesity Federation, Nutter and colleagues (2024) provide further reason to omit the word in most settings, highlighting the varied obesity definitions proposed by public health organizations[22]. For example, the World Health Organization (WHO) defines obesity as “abnormal or excessive fat accumulation that presents a risk to health” [23]. Conversely, the Centers for Disease Control and Prevention (CDC) defines obesity as a body mass index (BMI) greater than 30.0[24]. However, a 2022 position statement from the WHO challenges the CDC’s definition, stating that BMI-based approaches to obesity fail to acknowledge the location and function of adipose tissue, as it relates to adiposity-based chronic disease[23]. Additionally, Nutter and colleagues (2024) note that definitions of obesity that rely on BMI or other anthropometric metrics fail to capture obesity as a chronic disease[22]. A previous study supports this position, pointing out that weight and health are not always synonymous and that so-called adiposity-related health impairments can occur in those within the “healthy weight” range (when using BMI as a descriptor of one’s weight-related health)[25].

Given that the term “obesity” is poorly defined and is viewed as stigmatizing, testing modified versions of these measures is warranted. When searching for measures examining weight stigma toward others that use more neutral language descriptors (e.g., higher weight, larger-bodied), none were found. For internalized weight bias, it was noted that the Weight Bias Internalization Scale[26] had a modified version that changed the term "overweight" to "my weight," and maintained good reliability and validity[27]. Thus, the present study sought to adapt the Attitudes Towards Obese Persons (ATOP)[28] scale, a frequently used measure of weight stigma toward others. Though the ATOP is often used in current research, contemporary recommendations from major medical associations and peer-reviewed journals have suggested discontinuing the use of condition first language (e.g., "obese persons") in favor of people-first language (e.g., "persons with obesity";[29]). The original ATOP scale was compared to a modified version of the instrument, using neutral terms such as "higher weight." It was hypothesized there would be no difference in endorsed negative attitudes toward those with higher weight between those who completed the original ATOP compared to those who completed the modified version. Finally, given the original ATOP factor structure has been inconsistently replicated[30,31] since its validation study[28], the factor structure of both ATOP samples (ATOP original and ATOP modified) was examined. For example, researchers have found different two-factor models (suffer and inferior in a college sample; self-esteem and personality/social difficulties in a bariatric surgery sample)[30,31] In both of these studies, not all 20 ATOP items loaded onto a factor. Given the stigmatizing effects of terms like "obese," this study was conducted in hopes of gathering a better understanding of how weight-related terminology used in a research measure might relate to different attitudes towards individuals of higher weight.

## Methods

*Participants and Procedures*

Participants were recruited from a university subject pool at a midsized university in the northwestern region of the United States from November 2022 to December 2023. To be eligible to participate, participants needed to be at least 18 years or older. Participants completed a consent form at the beginning of the online Qualtrics survey and were able to receive course or extra credit for their participation. Alternative assignments were provided if students did not want to participate in research. For sample size for confirmatory factor analysis, the study aimed to obtain 10 participants per each of the 20 items. Thus, there were approximately 400 participants for each randomized group so data could be split into two randomized halves (approximately 200 participants in each condition) for the CFAs. Participants were then randomly assigned to complete either the original version of the Attitudes Toward Obese Persons Scale (ATOP)[28] or a modified version of the ATOP. After randomization, all participants completed additional questionnaires related to weight bias and eating disorder symptomatology.

*Measures*

**Demographics information**. Participants were asked to report age, gender identity, race, and ethnicity. Participants were asked to provide self-reported anthropometric information (height and weight), and then body mass index (BMI) was calculated. Total sample BMI ranged from 16.30 to 72.52 ($M$ = 24.99, $SD$ = 5.67), with 4.1% in the underweight BMI category, 57.5% in the healthly weight BMI category, 23.7%

in the overweight BMI category and 14.7% in the obesity BMI category. There were no significant differences between the ATOP group BMIs ($M$ = 24.92, $SD$ = 5.53) and ATOP-HW group BMIs ($M$ = 25.06, $SD$ = 5.79; $t(828) = -0.36$, $p$ = .720, CI [-0.91, 0.63]). Total sample age ranged from 18 to 52 ($M$ = 21.01, $SD$ = 5.40). There were no significant differences between the ATOP group ages ($M$ = 21.17, $SD$ = 5.69) and ATOP-HW group age ($M$ = 20.85, $SD$ = 5.09; $t(827) = 0.86$, $p$ = .389, CI [-0.41, 1.06]). Additional participant demographics for the randomized groups can be found in Table 1.

**Attitudes Toward Obese Persons Scale (ATOP[28])**. Negative stereotypical attitudes about people with obesity were measured using the ATOP. The ATOP is a 20-item measure using a Likert rating scale ranging from "I strongly disagree" (-3) to "I strongly agree" (3). A total score is calculated by reverse scoring the 13 negative items, summing the items, and then adding 60. Scores range from 0 to 120, where higher scores indicate more positive attitudes towards people with obesity. There are also three subscales, Different Personality (negative attributes about one's personality and abilities), Social Difficulties (experiencing social and interpersonal problems), and Self-Esteem. The ATOP has shown adequate internal reliability in adult populations[28]. In the current study, the ATOP had a McDonald's Omega of .84 and Cronbach's alpha of .84, demonstrating good internal consistency.

**Attitudes Toward Persons with Higher Weight; ATOP-HW)**. In the modified version of the ATOP, the term "obese" was replaced with "higher weight" or "larger body" and "nonobese" was replaced with "thin." The ATOP-HW had a McDonald's Omega of .82 and Cronbach's alpha of .82, demonstrating good internal consistency. See Table 2 for a comparison of wording between the two versions of the ATOP.

*Analytic Plan*

Analyses were conducted in IBM® SPSS® Statistics 29 and R. Descriptive statistics of participant demographics and internal consistency of the measures were examined. The sample was randomly split in half so that the factor structure determined in a principal component analysis (PCA) for the new ATOP-HW for the first half of the data could be confirmed in a CFA with the second half of the data. First, the two ATOP randomized groups were compared on their total ATOP scores using an independent-sample t-test. Next, two confirmatory factor analyses (CFA) were conducted in R[33] with package lavaan[34]. Model fit was evaluated by the following model fit indices: the comparable fit index (CFI; at least 0.95), the standardized root-mean square error of approximation (RMSEA; 0.06 or below), and the standardized root-mean square residual (SRMR; 0.08 or below)[35,36]. Following the findings of the CFAs, PCA were conducted for the ATOP-HW to determine a suitable factor structure, and for the original ATOP to compare factor structures. Finally, another CFA was conducted, this time on the second half of the ATOP-HW data, to verify the factor structure indicated in the PCA. Reliability statistics are also presented for the modified measures. The university Institutional Review Board approved the study procedures (#185-21).

## Results

There were 884 students who consented to participate in the study. Of these, three were excluded due to being under the age of 18, and 22 were removed due to missing the survey data needed for the study. Attention checks were included throughout the survey (e.g., “select always”). Of the remaining participants, 27 failed at least two of the three attention checks and, as such, were removed. A total of 832 participants remained for analyses.

An assessment of missing data was completed. For the original ATOP, three participants missed one to three items. Their missing items were replaced with the case mean[37]. Data was assessed for normality, and no data required transformation or removal.

To assess whether the use of non-stigmatizing language affected ATOP scores, aggregate scores were calculated for participants who received the original ATOP (*N* = 202, *M* = 69.25, *SD* = 16.49) and those who received the modified version (*N* = 214, *M* = 72.85, *SD* = 15.84). Contrary to the primary hypothesis, there was a statistically significant difference between the scores of participants who received the original version (ATOP) and those who received the modified version (ATOP-HW), $t(414) = -2.27$, $p = .024$ (two-tailed), 95% CI [-6.71, -0.48]. The effect size was small as calculated by Cohen’s $d = -0.22$, and very small as calculated by eta squared = .006.

Next, half the cases were randomly selected to be included in two confirmatory factor analyses (CFAs) to determine if the factor structures of the ATOP-HW and ATOP samples were consistent with the original ATOP factor structure[28]. In the original study, Allison and colleagues (1991)[28] identified a three-factor structure (Different Personality, Social Difficulties, and Self-Esteem). In both CFAs conducted, items were specified to load only on the items’ first-order latent factor. A poor model fit was found for the original ATOP, CFI = 0.767, TLI = 0.735, SRMR = 0.082, RMSEA = 0.080, 90% CI [0.070, 0.091], $\chi2$ (167, *N* = 202) = 385.13, p < .001. A poor model fit was also found for the ATOP-HW, CFI = 0.746, TLI = 0.711, *SRMR* = 0.078, *RMSEA* = 0.078, 90% CI [0.068, 0.089], $\chi2$ (167, *N* = 214) = 386.87, p < .001.

Given the poor model fit for the three-factor solution, the commonly used unidimensional approach with the original ATOP[38], and recent studies finding two-factor model fits[30,31], principal component analyses (PCAs) were conducted to determine an acceptable factor structure for the ATOP-HW. Data was deemed suitable for PCA, with numerous coefficients in the correlation matrix above .3, Kaiser-Meyer-Olkin of .818[39], and a significant Bartlett’s Test of Sphericity ($p < .001$)[40]. Five components were identified with eigenvalues > 1, which explained 24.3%, 9.7%, 7.8%, 6.4%, and 5.7% of the variance in the data. However, a clear break in the screeplot was identified after the first factor, most items cross-loaded with component one, and one included less than three items on the component. As such, a unidimensional solution was explored for the ATOP-HW, which is also in line with how most research currently uses the original ATOP. The unidimensional solution explained 24.3% of the variance in the data. Only eight of the 20 items had communalities of at least .30, indicating at least fair fit[32]. The brief 8-item ATOP-HW maintained acceptable internal consistency (McDonald’s Omega of .79; Cronbach’s alpha of .79) and the items maintained and removed made theoretical sense (see Table 3 for factor loadings). When confirming the 8-item ATOP-HW in another PCA, the unidimensional solution explained 40.6% of the variance in the data, with all communalities above .30 and factor loadings above .45.

A PCA was also conducted on the original ATOP to determine if the ATOP-HW and ATOP factor structures mirror each other. Data was deemed suitable for PCA, with numerous coefficients in the correlation matrix above .3, Kaiser-Meyer-Olkin of .814[39], and a significant Bartlett's Test of Sphericity ($p < .001$)[40]. Again, five components were identified with eigenvalues > 1, which explained 25.5%, 10.0%, 8.4%, 6.0%, and 5.3% of the variance in the data. Like with the ATOP-HW, a clear break in the screeplot was identified after the first factor and most items cross-loaded with component one. As such, a unidimensional solution was explored for the ATOP, which is also in line with how most research currently uses the original ATOP. The unidimensional solution explained 25.5% of the variance in the data. Nine of the 20 items had communalities of at least .30, indicating at least fair fit[41]. The ATOP retained one extra item than the ATOP-HW. Using the same 8-items retained in the ATOP-HW PCA, the brief 8-item ATOP maintained good internal consistency (McDonald's Omega of .82; Cronbach's alpha of .82).

Finally, with the second half of the randomly split dataset, a CFA was conducted on the new 8-item ATOP-HW to verify the brief, 8-item factor structure in this second dataset. Items were specified to load only on the items' first-order latent factor. The 8-item ATOP-HW met the requirements for each of the fit indices, indicating the model fit the data well, CFI = 0.978, *SRMR* = 0.042, *RMSEA* = 0.045, 90% CI [0.000, 0.081], $\chi 2$ (20, $N$ = 199) = 28.14, $p = .106$.

## Discussion

The present study aimed to examine the implications of changing the language used to describe individuals in the ATOP measure from "obesity/obese" to "higher weight." The study sought to revise potentially stigmatizing language within a commonly used measure of weight stigma while retaining reliability and validity. The results showed that there was a statistically significant difference in endorsement of weight-based stigma between the two versions of the ATOP (original and modified). Specifically, participants who were given the version of the ATOP with less stigmatizing language (i.e., higher weight) endorsed slightly less stigmatizing beliefs than individuals who completed the original version (i.e., obese/obesity). It is possible that using less stigmatizing language results in slightly lower weight stigma. However, the practical significance of the differences was very small or small depending on effect size indices, and the ATOP-HW mean was similar to ATOP means found in previous studies with student participants[e.g.,42]. Internal consistency was found to be good for the ATOP-HW. These findings provide support for a revised version of ATOP used to measure negative attitudes toward those with higher weight, without greatly sacrificing psychometric properties, but potentially capturing slightly lower endorsement of weight stigma than the traditional ATOP.

Researchers should further examine how participants are conceptualizing the individuals described in weight stigma measures. Future research should focus on whether any size or weight-related terms (e.g., obese, higher-weight, fat) are interpreted differently for some respondents. Instead of capturing feelings about individuals living in larger bodies, participants may be instead endorsing their feelings towards individuals with metabolic conditions who are living at higher weights. Their stigmatizing beliefs may be influenced by the assumptions made about the health of the patients described rather than being solely influenced by their body size or appearance. There is not a clear consensus on the definition of obesity; it is sometimes defined as having a BMI above 30, it is sometimes defined as a disorder involving excess adiposity or body fat, and it is sometimes defined as a metabolic condition[43]. The conflation of health and weight within many of the definitions may be similarly reflected in their endorsements of stigmatizing beliefs.

The original and modified versions of the ATOP also performed similarly in an examination of their factor structure. The three-factor structure (Different Personality, Social Difficulties, and Self-Esteem) identified in the original ATOP validation study was not replicated in either sample (original or modified ATOP). Instead, a briefer, unidimensional structure was found that retained eight of the 20 items on the modified ATOP-HW. These findings are consistent with existing literature as very few studies have successfully replicated the original ATOP three-factor structure[28]. Of note, most studies already use the ATOP as a unidimensional measure of weight-stigmatizing attitudes[37], despite the original three-factor structure, which our findings support.

In looking at the items removed from the unidimensional modified ATOP, these removed items mostly required respondents to assume the internal feelings or perspectives of those with higher weight (e.g., “Very few higher-weight people are ashamed of their weight,” “Most higher-weight people feel that they are not as good as other people”). It may be that these internal self-esteem items do not consistently fit the other weight stigma items that focus more on negative perceptions of personality and attributes (e.g., “People with very high weight are usually untidy,” “People in larger bodies are just as sexually attractive as thin people”). Indeed, the items retained make theoretical sense, and the brief, 8-item ATOP-HW appears a face-valid measure for contemporary negative attitudes toward those with higher weight without making assumptions about the internal feelings of others and reducing burden for participants completing the ATOP-HW. Importantly, the follow-up CFA verified the brief, 8-item factor structure in the second dataset, finding the model fit the data well. Future research should continue refining the field’s measurement of weight stigmatizing attitudes, especially as these attitudes may transform with cultural changes throughout time.

Given the stigma of the term“obesity” identified by people with lived experience[16,17] and the inconsistent definitions and uses of “obese” and “obesity”[22,43], it may be useful for researchers to refine existing measurements of anti-fat weight bias to a more descriptive, neutral term to describe people’s body size. Of note, our examination of revised language includes just some terminology options, and there may be variability among people with lived experience of higher weight in terms of preferred terms (e.g., fat, higher-weight, etc.). As language and perceptions about weight evolve through time, continued exploration of psychometrically sound yet inclusively worded research measures is indicated. Though scores on the ATOP and ATOP-HW were statistically different, with the small effect size, it is unknown whether the different endorsements of weight stigma are practically meaningful in the real world. Peer-reviewed journals and health organizations are encouraging researchers and health care providers to differentiate between higher-weight and higher-weight with adverse health outcomes [(e.g., *21;* 23)], yet the field knows little about how changing these terms may change the level of negative attitudes towards people with higher weight. If all it took to significantly reduce the endorsement of stigma was to swap out the language used to describe patients, then this speaks to the potential utility of interventions in the healthcare domain that push for not only person-first language, but also the replacement of the word obesity with a neutral descriptor of size instead. More longitudinal research and more intervention studies are needed to understand whether the change of language can have significant and enduring impacts on the endorsement of stigma and stigmatizing beliefs. Future research should consider using qualitative or mixed-methods research to better understand the definitions of each of these constructs in the general population and how these relate to stigmatizing beliefs about those with higher weight.

Though this research has important implications, there are several limitations to acknowledge and address in future research. First, our sample is a generally age-restrictive, majority White college student population, which could impact generalizability. Though a range of weight distribution was represented in the sample, the proportion of people with higher weight was lower than the United States average[44]. Future research should consider validating the ATOP-HW in other populations, including healthcare professionals, individuals in larger bodies, and diverse community samples to assess measurement invariance across populations. Further, this study was also strictly quantitative, which means it is unknown how participants conceptualized the individuals described in the measures. Future research should focus on whether any size or weight-related terms (e.g., obese, higher-weight, fat) are interpreted differently for some respondents. Further examination of the terminology used in research contexts is needed to determine how to best assess weight stigma without reinforcing stigmatizing attitudes.

## Conclusion

In conclusion, this study provides initial support for modernizing the ATOP by shifting use to a brief, 8-item ATOP-HW that uses neutral language to describe body size. As researchers aiming to understand and document the harms associated with weight-related bias and discrimination, it is crucial that researchers do not perpetuate the stigma through what measures are being used in the research. The findings of the present study suggest that the use of neutral terms in measures of anti-fat bias is a promising solution that warrants further investigation. It is vital that scholars remain critical of research methods and measures, reaffirming their relevance and validity as cultural norms about language and weight continue to change. The present study represents an important first step in this process.

## Declarations

Ethics approval and consent to participate: The study was conducted following the Declaration of Helsinki and was approved by the Institutional Review Board at the University of Montana (#185-21).

Disclosure: The authors declare no conflict of interest.

Data Availability Statement: The datasets generated and/or analyzed during the current study are available in the Open Science Framework repository, https://osf.io/cn7fv.

Funding: This research was supported by the National Institute of General Medical Sciences of the National Institutes of Health Award Number P20GM130418.

CRediT: CMW-conceptualization, data curation, formal analysis, funding acquisition, investigation, methodology, project administration, resources, software, supervision, validation, visualization, writing original draft, writing review and editing; KM-data curation, formal analysis, visualization, writing original draft; SA-data curation, formal analysis, writing original draft; BB-conceptualization, methodology, visualization, writing original draft, writing review and editing; CT-investigation, writing original draft; OK investigation, writing original draft; TP visualization, writing original draft. All authors read and approved the final manuscript.

## Tables

**Table 1**

*Demographic Characteristics*

| Characteristic | Total Sample $N$ = 832 $n$ | % | ATOP Sample $n$ = 419 $n$ | % | ATOP-HW Sample $n$ = 413 $n$ | % |
|---|---|---|---|---|---|---|
| Gender Identity | | | | | | |
| Cisgender woman | 608 | 73.1 | 310 | 74.0 | 298 | 72.2 |
| Cisgender man | 191 | 23.0 | 90 | 21.5 | 101 | 24.5 |
| Nonbinary | 19 | 2.3 | 12 | 2.9 | 7 | 1.7 |
| Genderfluid | 7 | 0.8 | 5 | 1.2 | 2 | 0.5 |
| Transgender man | 4 | 0.5 | 3 | 0.7 | 1 | 0.2 |
| Transgender woman | 3 | 0.4 | 3 | 0.7 | 0 | 0.0 |
| Demi-girl | 2 | 0.2 | 1 | 0.2 | 1 | 0.2 |
| Agender | 1 | 0.1 | 1 | 0.2 | 0 | 0.0 |
| Questioning | 1 | 0.1 | 1 | 0.2 | 0 | 0.0 |
| Prefer not to say | 3 | 0.4 | 1 | 0.2 | 2 | 0.5 |
| Race/ethnicity | | | | | | |
| European American/White | 739 | 88.8 | 375 | 89.5 | 364 | 88.1 |
| Native American/American Indian/Alaska Native/Indigenous | 65 | 7.8 | 30 | 7.2 | 35 | 8.5 |
| Asian/Asian American | 40 | 4.8 | 18 | 4.3 | 22 | 5.3 |
| Hispanic/Latino/a/x | 50 | 6.0 | 27 | 6.4 | 23 | 5.6 |
| African American/Black | 13 | 1.6 | 9 | 2.1 | 4 | 1.0 |
| Pacific Islander | 10 | 1.2 | 6 | 1.4 | 4 | 1.0 |
| Middle Eastern/North African | 5 | 0.6 | 2 | 0.5 | 3 | 0.7 |
| Unknown | 3 | 0.4 | 1 | 0.2 | 2 | 0.5 |
| Sexual Orientation | | | | | | |
| Straight/Heterosexual | 617 | 74.2 | 310 | 74.0 | 307 | 74.3 |
| Bisexual/Pansexual | 157 | 18.9 | 82 | 19.6 | 75 | 18.2 |
| Asexual | 29 | 3.5 | 14 | 3.3 | 15 | 3.6 |
| Lesbian/Gay | 22 | 2.6 | 9 | 2.1 | 13 | 3.1 |
| Queer | 2 | 0.2 | 2 | 0.5 | 0 | 0.0 |
| Non-Labeled | 1 | 0.1 | 1 | 0.2 | 0 | 0.0 |
| Questioning | 3 | 0.4 | 2 | 0.5 | 1 | 0.2 |
| Unknown | 2 | 0.2 | 0 | 0.0 | 2 | 0.5 |
| Year in College | | | | | | |
| Freshman/first year | 477 | 57.3 | 244 | 58.2 | 233 | 56.4 |
| Sophomore | 156 | 8.8 | 79 | 18.9 | 58 | 18.6 |
| Junior | 109 | 13.1 | 51 | 12.2 | 39 | 14.0 |
| Senior | 68 | 8.2 | 34 | 8.1 | 27 | 8.2 |
| 5th year and beyond | 22 | 2.6 | 11 | 2.6 | 9 | 2.7 |

*Total percentages for demographics are beyond 100% because participants could select more than one category.

**Table 2**

*Wording for the Original ATOP and Modified Version (ATOP-HW)*

| Original Version | Modified Version |
|---|---|
| 1. *Obese* people are as happy as *nonobese* people. | 1. *Higher-weight* people are as happy as *thin* people. |
| 2. Most *obese* people feel that they are not as good as other people. | 2. Most *higher-weight* people feel that they are not as good as other people. |
| 3. Most *obese* people are more self-conscious than other people. | 3. Most *higher-weight* people are more self-conscious than other people. |
| 4. *Obese* workers cannot be as successful as other workers. | **4. *Higher-weight* workers cannot be as successful as other workers.** |
| 5. Most *nonobese* people would not want to marry anyone who is *obese*. | **5. Most *thin* people would not want to marry anyone who has a *larger body*.** |
| 6. Severely *obese* people are usually untidy. | **6. People with *very high weight* are usually untidy.** |
| 7. *Obese* people are usually sociable. | 7. *Higher-weight* people are usually sociable. |
| 8. Most *obese* people are not dissatisfied with themselves. | 8. Most people with *higher-weight* are not dissatisfied with themselves. |
| 9. *Obese* people are just as self-confident as other people. | 9. People with *higher-weight* are just as self-confident as other people. |
| 10. Most people feel uncomfortable when they associate with *obese* people. | 10. Most people feel uncomfortable when they associate with people with *higher-weight*. |
| 11. *Obese* people are often less aggressive than *nonobese* people. | 11. *Higher-weight* people are often less aggressive than *thin*. |
| 12. Most *obese* people have different personalities than *nonobese* people. | 12. Most *larger bodied* people have different personalities than *thin* people. |
| 13. Very few *obese* people are ashamed of their weight. | 13. Very few *higher-weight* people are ashamed of their weight. |
| 14. Most *obese* people resent normal weight people. | 14. Most *higher-weight* people resent *thinner* people. |
| 15. *Obese* people are more emotional than *nonobese* people. | **15. People with *higher-weight* are more emotional than *thin* people.** |
| 16. *Obese* people should not expect to lead normal lives. | **16. People with *higher-weight* should not expect to lead normal lives.** |
| 17. *Obese* people are just as healthy as *nonobese* people. | 17. People with *higher-weight* are just as healthy as *thin* people. |
| 18. *Obese* people are just as sexually attractive as *nonobese* people. | **18. People in *larger bodies* are just as sexually attractive as *thin* people.** |
| 19. *Obese* people tend to have family problems. | **19. People in *larger bodies* tend to have family problems.** |
| 20. One of the worst things that could happen to a person would be for him to | **20. One of the worst things that could happen to a person would be for *them* to** |

| | |
|---|---|
| become *obese*. | **become *higher weight*.** |

*Note.* Modified language is shown in italics for ease of the reader. ATOP = Attitudes Toward Obese Persons Scale.
Items in bold were retained in the brief, final version of the Attitudes Towards People with Higher Weight Persons Scale (ATOP-HW)

**Table 3**

*ATOP-HW Factor Loadings*

| Item | Factor Loading |
|---|---|
| 6. People with very high weight are usually untidy. | .66 |
| 5. Most thin people would not want to marry anyone who has a larger body. | .63 |
| 18. People in larger bodies are just as sexually attractive as thin people. | .62 |
| 4. Higher-weight workers cannot be as successful as other workers. | .59 |
| 19. People in larger bodies tend to have family problems. | .58 |
| 15. People with higher-weight are more emotional than thin people. | .57 |
| 16. People with higher-weight should not expect to lead normal lives. | .56 |
| 20. One of the worst things that could happen to a person would be for them to become higher weight. | .56 |
| 1. Higher-weight people are as happy as thin people. | .54 |
| 10. Most people feel uncomfortable when they associate with people with higher-weight. | .53 |
| 12. Most larger bodied people have different personalities than thin people. | .52 |
| 9. People with higher-weight are just as self-confident as other people. | .51 |
| 17. People with higher-weight are just as healthy as thin people. | .48 |
| 3. Most higher-weight people are more self-conscious than other people. | .45 |
| 2. Most higher-weight people feel that they are not as good as other people. | .43 |
| 14. Most higher-weight people resent thinner people. | .42 |
| 8. Most people with higher-weight are not dissatisfied with themselves. | .22 |
| 11. Higher-weight people are often less aggressive than thin. | .21 |
| 7. Higher-weight people are usually sociable. | .12 |
| 13. Very few higher-weight people are ashamed of their weight. | .01 |
